# Judicial outcome and follow up of abused child protection acts in a pediatric emergency department: 12-year experience in a third level pediatric hospital

**DOI:** 10.1186/s13052-020-00823-6

**Published:** 2020-05-13

**Authors:** Federico Poropat, Arianna Canuto, Giulia Caddeo, Erica Predonzani, Laura Novello, Alessandra Zorzetto, Claudio Germani, Egidio Barbi

**Affiliations:** 1 Institute for Maternal and Child Health IRCCS Burlo Garofolo, Via dell’Istria 65/1, 34137 Trieste, Italy; 2 University of Trieste, Trieste, Italy; 3 Social worker, Trieste district, Italy

**Keywords:** Child protection, Out-of-home care, Family Reunion, Development trajectory in abuse child

## Abstract

**Background:**

Italian laws allow the protection of a child who is suspected to be a victim of abuse through a procedure that can be put in motion by the attending physician in any Emergency Department (article nr. 403 Civil Code) with a temporarily suspension of parental authority.

This study aims at evaluating both the appropriateness of the activation of the protection procedure by ED doctors in cases of suspected child abuse in terms of judicial confirmation and how it impacts children in the long-term.

**Methods:**

We selected cases in which the procedure was activated due to suspected child abuse. The children were admitted to the ED of a tertiary children hospital from 2006 to 2018. We then reviewed the medical charts and collected data from the social services through a questionnaire concerning the long-term outcomes .

**Results:**

Twenty-eight patients were included (75% females, mean age 13.8 years). In 90% of cases the activation of the procedure in the ED was followed by a Court confirmation. Evaluation of long term outcome was possible in 22 cases. Among them, a positive social outcome was achieved in 15 cases (68.2%). The remaining abandoned the program or had critical reintegration in the family. Eighteen percent of patients developed major issues such as aberrant behaviours, substance abuse or psychiatric disorders.

**Conclusions:**

This report identifies a good ability of ED doctors in the activation of an emergency procedure to protect the child. Overall, the social outcome was good for nearly 70% of the patients, highlighting the importance of activation of social support programs for child abuse.

## Background

In 1999, the World Health Organization (WHO) defined child abuse as “all forms of physical and/or emotional ill-treatment, sexual abuse, neglect or negligent treatment or commercial or other exploitation, resulting in actual or potential harm to the child’s health, survival, development or dignity in the context of a relationship of responsibility, trust or power” [1].

Child abuse is an underestimated issue due to the lack of reports by the victims themselves and the difficulty in recognizing and intervening on the part of health professionals.

According to the “Global Status Report on Violence Prevention”, published in 2014 by WHO, one adult in four undergoes physical abuse as a child.

In Italy it is estimated that there are at least 91,000 victims of abuse under the age of 18, with violence mostly occurring in the domestic sphere [2].

The consequences of child abuse include not only immediate injuries, as in the case of physical abuse, but also severe late repercussions on neurologic, cognitive and emotional development of the child. This is also the case for psychological abuse, assisted violence or neglect.

In these situations, a prompt recognition and intervention have proven to reduce the impact on the child.

As stated by article 403 of the Italian Civil Code any ED physician suspecting child abuse can put the safety procedure in motion if the family environment cannot be considered safe.

With this emergency procedure, the parental authority may be temporarily suspended and the child removed from the unsafe environment and relocated to in a safe one, usually under the guardianship of a protective family member, whenever it can be found, or a residential child care community.

The provision lasts until the end of the investigations by the Competent Court, which may decide to confirm or deny the need for child’s protection. In case of confirmation, the parental authority will remain suspended and the victim protected until the family demonstrates to be able to properly take care of him/her appropriately or, if this is not possible, until the child turns 18, which by Italian law is the age of adulthood.

This study aims at evaluating both the appropriateness of activation of this child’s protection procedure (article nr. 403 c.c.) by ED doctors in terms of judicial confirmation and how it impacts children in the long-term.

## Methods

We conducted a retrospective study analysing all the cases of suspected child abuse that resulted in the activation of the child’s protection procedure (article nr. 403 c.c.) by doctors of the Pediatric Emergency Department of the “Institute for Maternal and Child Health IRCCS Burlo Garofolo” in Trieste, Italy. The Pediatric ED is part of a third level pediatric teaching hospital, serving an area of 250.000 people, with an average of 25.000 admissions of patients up to 18 years of age each year.

The study was approved by the Local Ethical Committee.

The study-period considered stretches from January 2006 (year in which a multidisciplinary protocol for the investigation of abused and neglected children - see Additional file 1) till April 2018.

Cases were identified through the ED database. For each of them historical, demographic and medical data were collected.

The list of the collected cases was then shared with the social workers (*Third District – ASL1 Triestina - Valmaura*) who are in charge of managing these situations and who are the only ones authorized by the Italian Law to consult children’s data after their discharge from the hospital.

A questionnaire concerning the short and long-term outcomes of the activation of the procedure mentioned above was given to the social workers. In particular, this included questions about the confirmation or denial of the parental authority suspension by the Competent Court, about who took care of the minor after the activation of the article nr. 403, how long the child’s protection lasted, and where the child was placed at the end of the program.

The primary outcome chosen for this study was the number of activations of the protection procedure according to the article 403 consequently confirmed by the Competent Court. This was considered as a proxy measure of doctors’ appropriateness in recognizing situations of potential danger for the child since it demonstrated the appropriateness of the medical intervention in recognizing situations of the potential danger for the child.

The secondary outcomes concerned the main long-term social and health consequences of this procedure for the children and their families. More precisely, assuming that the activation of the procedure allowed to protect the children from potentially dangerous situations and to recover their right to a healthy growth, we arbitrarily defined as a positive result both a successful reintegration in the original family or the permanence in the assigned Residential Child Care Community. The personal and social outcome (school attendance) was defined according to the records of the educators and social workers, until children reached the age of majority.

On the other hand, we arbitrarily considered all the cases where the reintegration into the family failed, the child abandoned the project, developed behavioral problems, presented with psychiatric disorders, alcohol and drug abuse, or loss of school attendance as negative results.

### Statistical analysis

We performed the statistical analysis using the website OpenEpi19.

We expressed the continuous data as means and range, the categorical data as absolute frequencies and percentages, and performed comparisons using Student’s t-test or Fisher’s exact test for continuous and categorical variables, respectively. Statistical tests were two-sided, and a *p*-value of less than 0.05 was considered statistically significant.

## Results

During the 12-years study period, 371 patients were referred from the ED to the social workers in the suspicion of child abuse; among them, for 32 cases (8.6%) the ED doctor decided to activate the emergency procedure (article nr. 403 c.c.) in order to provide immediate protection to the child.

Four cases were excluded from the analysis because data from social work services were incomplete.

The remaining 28 patients were included in the study; their characteristics and the types of abuse for which the emergency procedure was activated are summarized in Table 1.

**Table 1:** Characteristics of the patients included in the study and types of abuse for which the emergency procedure was activated

	N (%)
**Gender,**	
Male	7 (25)
Female	21 (75)
**Type of abuse**	
Physical/emotional abuse	18 (64.3)
Neglect	7 (25)
Sexual abuse	3 (10.7)
**Mean age in years at 403 activation (range**)	13.8 (1.7–17)
**Adolescents – older than 14 year-old -** (%)	18 (64.3)

The Competent Court confirmed the suspicion of abuse and the need for the child’s protection in 25 out of the 28 patients (89.3%).

All the patients were removed from their families, at least for the time requested by the Competent Court to complete the investigations.

The length of stay in out-of-home placement was variable, with a mean of 15.8 months (range 1 month – 5 years). Sixteen children (57,8%) re-entered in their own family in less than 12 months. No time difference was found considering the cause of abuse or the age groups (more or less than 14 years old).

Twenty-six children (92.9%) were sent to a Residential Child Care Community; in two cases child custody was granted to other relatives (grandparents). The mean age of patients in kinship care was younger (5.4 years versus 14.4 years, *p* 0.05) and the reintegration in the original family faster (4.5 months versus 16.6 months), but the difference was not statistically significant.

**Fig. 1:**
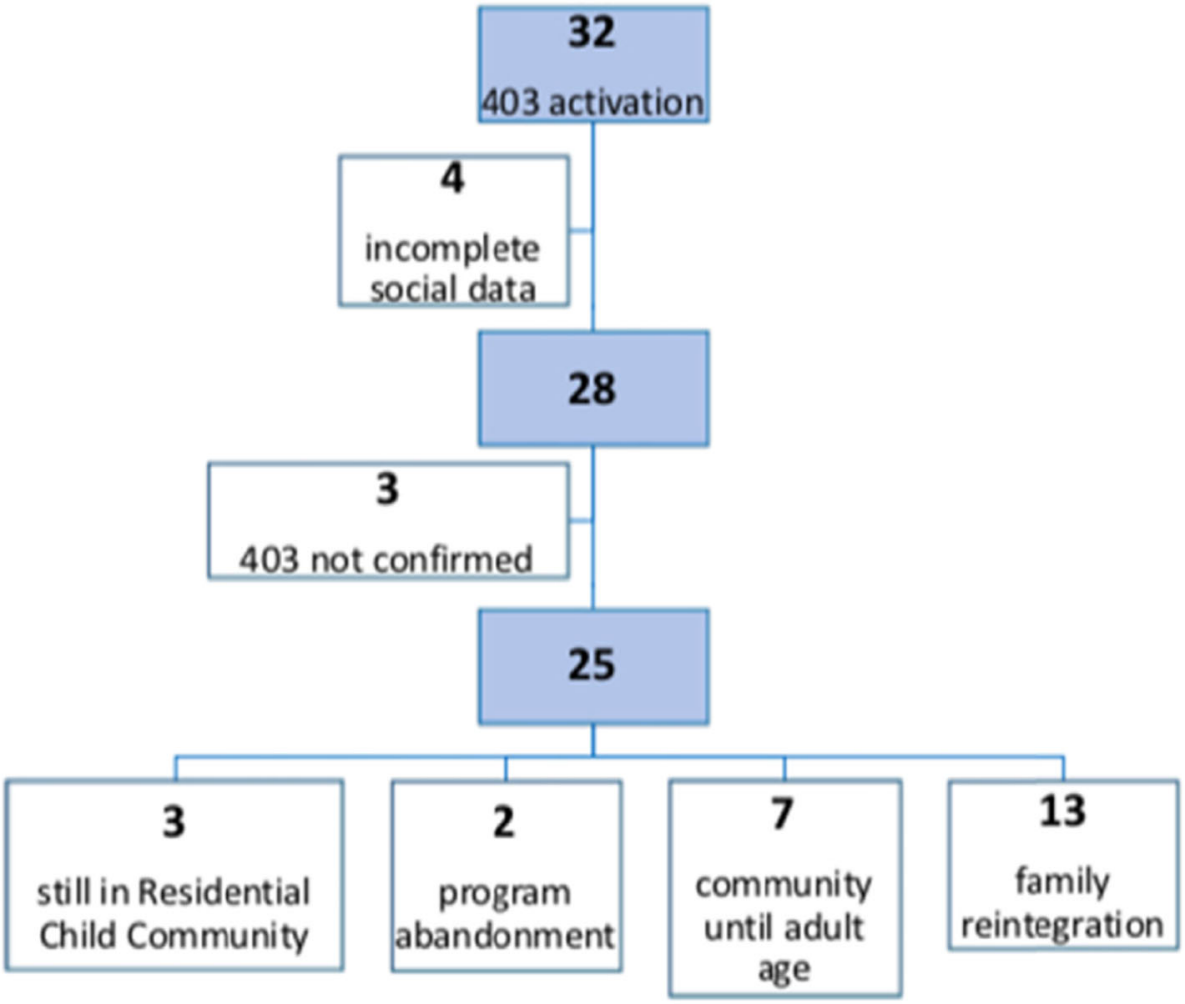
Outcome of the 28 patients included in the study, data collected from the social services charts

Regarding the secondary outcomes (Fig. 1), among the 25 children to whom the Competent Court confirmed the suspension of parental authority, three minors did not complete the social counselling pathways before the end of the study and were therefore excluded from the analysis. They were three patients older than 14 years old. Of the remaining 22 cases, two girls (9.1%) voluntarily abandoned the Residential Community assigned and the social program. Seven adolescents (31.8%), with a mean age of 15.7 years (range 14–17), remained in the Residential Child Care Community until the adult age with positive outcome. Thirteen patients (59.1%) were reintegrated in the original families.

Among this last group, the reintegration was entirely positive for eight (61.5%), while for the other five (38.5%), despite the conclusion of the social program, the reintegration was at some points critical, due to remaining issues concerning parents, children or both.

A positive social outcome was achieved in 15 case (68.2%) including children who were successfully reintegrated into the original family and those who remained in the Residential Child Community completing the program.

Seven cases failed to reach the target (31.8%), considering adolescents who abandoned the social program and those which had a critical reintegration into the original family.

Focusing on the health outcome, 4 cases (18.2%) developed specific diseases, such as substance abuse, psychiatric disorders or aberrant behaviours and in particular three of them had more than one disturb. None of those who were reintegrated in their families successfully developed a comorbidity.

In Table 2 the positive outcome group is compared to the negative outcome group: no significant differences were found between them, except for the development of comorbidities, that only occurred only in the negative one.

**Table 2:** Comparison between the positive and negative outcome groups

	Positive outcome (%)	Negative outcome (%)	*p-values*
Gender, N (%)			
Male	4 (26.7)	1 (14.3)	
Female	11 (73.3)	6 (85.7)	0.954
Age at 403 activation (years), mean (range)	14.9 (9–17)	13.7 (7–17)	0.38
Adolescents (age 14 and over), N (%)	12 (86.7)	4 (57.1)	0.534
Type of abuse, N (%)			
Physical/emotional abuse	10 (66.7)	5 (71.4)	0.999
Neglect	2 (13.3)	2 (28.6)	0.756
Sexual abuse	3 (20)	0 (0)	0.591
Permanency in out-of-home placement (months), mean (range)	17.9 (1–60)	15 (3–24)	0.70
Permanency in out-of-home placement 1 year or less, N (%)	8 (53.3)	4 (57.1)	0.999
Development of comorbidities, N (%)	0 (0)	4 (57.1)	*p* 0.009

*p*-values > 0.05 are not statistically significative

## Discussion

This paper shows that the overwhelming majority of the emergency procedures, for the protection of abused children, activated by ED pediatricians was deemed appropriate by the Competent Court, confirming the ability of the medical staff to recognize seriously compromised situations and using appropriate legal and social tools. The downside of this aspect is that some families were separated due to the overly intrusive welfare and medical system. However, as doctors, we have to bear in mind two decisive facts. Firstly, the separation of the child from his parents represents an extrema ratio of the intervention of the welfare state, which involves only few children who seems to be in an extremely dangerous situation. Secondly, clinicians must pay close attention to suspected child maltreatment to avoid outcomes that, if not recognized, could be dangerous and even life-threatening. Indeed the current epidemiology of abused children shows that the majority of victims are not identified, and the size of the problem, as we perceive, is more significant than previously thought. Neglected or abused children, if they survive abuse, have an altered trajectory of brain development, affecting sensory systems, network architecture, circuits involved in threat detection, emotional regulation, and reward anticipation. Two meta-analyses conducted in 2003 demonstrate that exposition of children to domestic violence was related to emotional and behavioural problems [1, 3], resulting in internalizing (e.g., depression, low self-esteem, and withdrawal), or externalizing (e.g., rebellion, hyperactivity, and delinquency) outline [4]. Not secondarily, being a victim of violence is one of the main risk factors to became an abuser, in turn, widening and perpetuating the problem. Therefore, as the International Society for the Prevention of Child Abuse and Neglect suggests, even the mere suspicion of maltreatment is sufficient to activate the social protection system to help parents develop more effective parenting skills and to improve overall functioning. Family preservation is the first social intervention that seeks to reduce out-of-home placement, but it is not always available in tight deadlines, and removal can be the safest temporary measure.

Unlike other European experiences [5], residential care institutions represent the most common out-of-home resource in our cohort, and in this series, only 8% of children were left with a relative’s family. In any case, the foster care collocations were not started because the Italian welfare system does not allow a rapid activation of this kind of institution. Previous studies showed that entrusting children to the care of relatives represents the measure with the best impact on their behavioral well-being, benefiting from a higher psychological stability [6, 7] and a reduction in the risk of changing placements. Unfortunately, in our experience, the relatives of the victims are rarely suitable to take care of them, preventing social workers from taking this path. Except that for the younger age there are no differences related to demographic aspects or type of mistreatment in children entrusted to relatives. The size of the cohort does not allow us to distinguish each group in terms of outcome.

Our study demonstrates that the length of staying out-of-home is variable, depending on the severity of social problems and collaboration obtained from child and family. However more than half of children came back to the biological family within 12 months, similarly as reported by US department in the American experience [8].

The time needed before the re-collocation of the child in the familiar environment is secondary to the availability and receptivity of the parents; however, several studies that compared the children reunited with their families with those who remained in care of social structures have led to contradictory conclusions on the risks of emotional problems, self-harming, and dangerous behaviours [9–12].

Overall, almost 2/3 of children came back to the biological family and reached a positive trajectory with successful reintegration. The fact that most of the children went back to their birth parents after placement reflects the central importance of reunification as an outcome of out-of-home placement. The children with a reunited trajectory interrupted with the need to return to out-of-home care were younger than the others and were all victims of physical and neglected abuse. We did not find difference in the time of separation, age at first out-of-home allocation, or sex. Some studies on the out-of-home care showed that reunification fails in almost 22–33% of the cases causing a re-entering of the child in the social care protection [13–15]. Others tried to outline likely risk factors due to the lack of reunification or the need to re-entry in the out-of-home care after a failed reunion, showing opposing results. The older age seems to be a factor influencing reintegration into the original family. Indeed the children who remained in the Residential Child Care Community were all over 16 years old, but all with good outcomes. The parent unemployment status, a teenage mother, more than the age of father, is correlated with a minor chance to reunify the original family [14].On the contrary, immigrant families and the existence of siblings seem to be associated with a higher likelihood of reunification [16, 17].

Overall, in the whole cohort, considering the successfully reunified families and adolescents never gone back to the family, 70% of children had a good social and well-being outcome. A generalization of these results is very difficult due to the unique and not always measurable factors, as the severity and length of the period of maltreatment experienced. Moreover, it is debated if the outplacement is really beneficial or inconsequential for the development and well-being of maltreated children [15, 17–19].

Focusing only on medical outcomes in terms of onset of new diseases, we note that in this series a successful reunion of the family is correlated to the well-being behavior of all the children, while half of the children with negative outcomes developed psychiatric disturbs and often more than one co-morbidity. This evidence has already been reported, showing as out-of-home placement “per sè” may represent a significant risk factor to develop psychiatric, psychological and criminal behaviors [18, 20].

To our knowledge, this is the first Italian study addressing the issue of abused children with out-of-home collocation started in the emergency department, with particular attention to the follow-up and long-term outcomes.

This study has some weaknesses. The limited number of cases does not allow us to achieve a firm conclusion, the follow-up data were available only until 18 years old, preventing us from discussing the long-term outcome in adulthood, and for some patients the final follow-up lasted only few years. Further weaknesses are the limited data about the economic and ethnic conditions of the families involved in the study and the nature of the study that does not allow us to establish which are the correlations among the co-morbidity and the out-of-home care collocation.

## Conclusions

This report identifies the professional ability of ED doctors in the activation of the emergency procedure to protect the child with a 90% rate of eventual confirmation by the Competent Court. Overall, the social outcome was good for nearly 2/3 of the patients, underlining the importance of early activation of social support programs against child abuse.

## Supplementary information

**Additional file 1.** Multidisciplinary protocol for the investigation of child abuse and neglect.

## Data Availability

The datasets used and/or analysed during the current study available from the corresponding author on reasonable request.

## Abbreviation

ED: Emergency department
