# Supplementary material for: Judicial outcome and follow up of abused child protection acts in a pediatric emergency department: 12-year experience in a third level pediatric hospital
Source: Ital J Pediatr. 2020 May 13;46:59. doi: 10.1186/s13052-020-00823-6 (PMC7218557; doi:10.1186/s13052-020-00823-6)
Supplement: Supplementary file 1 — Additional file 1. Multidisciplinary protocol for the investigation of child abuse and neglect. [file 13052_2020_823_MOESM1_ESM.doc]

**Multidisciplinary protocol for the investigation of child abuse and neglect**

The protocol for detecting children with suspect abuse was drafted in 2006 following a collaboration among territorial and hospital social services and doctors of our institution skilled on the abused child.

The goals of this protocol have been

- to educate and periodically re-train all health figures working in our institution (nurses, paediatricians, orthopaedics, radiologists, gynaecologists) to recognize and mange cases of suspect child abuse

- to create a strong network between our hospital and territorial health services to foster a more rapid taking charge of the family in the mild cases of mistreatment

- to outline explicitly the legal pathway of management of clear case of abuse

The protocol can be activated by ED doctor in any moment during patient’s assessment.

It identifies two situations.

1. child with vague abuse signs (ambiguous physical examination or weak suspect of neglect or odd behaviours): the emergency doctor contacts the hospital social workers (which contact territorial social workers in turn) and the general practitioner of the child to better outline the social picture of the family. If any doubts persist, judicial authority is cautioned and the child is given in custody to a multidisciplinary team and colloacted in a safe place. Otherwise the history is reported in a dedicated database of the ED to follow up the child if new admissions in the hospital happens.
2. child with classic signs of abuse or if caregiver, or child himself, report direct or indirect mistreatment: specific diagnostic procedure to verify the hypothesis of abuse and properly correct proofs are performed. The emergency doctor contacts the hospital social workers and Judicial authority. Then all parts involved (emergency doctor, social worker and child neuropsychiatric – if needed ) discuss together and judge the seriousness of the case. If a protective member cannot be individuated within the family, parental authority is temporarily suspended by judicial authority.

When paternal authority is temporary suspended, the child is removed from the unsafe environment and collocated in a safe one, usually a Residential Child Care Community or kin. A multidisciplinary team composed mainly by social workers and psycologists starts to taking care of the child and the family.
